# DNA-PK as an Emerging Therapeutic Target in Cancer

**DOI:** 10.3389/fonc.2019.00635

**Published:** 2019-07-17

**Authors:** Ismail S. Mohiuddin, Min H. Kang

**Affiliations:** Cancer Center, Department of Pediatrics, Pharmacology and Neuroscience, School of Medicine, Texas Tech University Health Sciences Center, Lubbock, TX, United States

**Keywords:** DNA-PKcs, DNA-PK, DNA repair, chemotherapeutic target, *PRKDC*

## Abstract

The DNA-dependent protein kinase (DNA-PK) plays an instrumental role in the overall survival and proliferation of cells. As a member of the phosphatidylinositol 3-kinase-related kinase (PIKK) family, DNA-PK is best known as a mediator of the cellular response to DNA damage. In this context, DNA-PK has emerged as an intriguing therapeutic target in the treatment of a variety of cancers, especially when used in conjunction with genotoxic chemotherapy or ionizing radiation. Beyond the DNA damage response, DNA-PK activity is necessary for multiple cellular functions, including the regulation of transcription, progression of the cell cycle, and in the maintenance of telomeres. Here, we review what is currently known about DNA-PK regarding its structure and established roles in DNA repair. We also discuss its lesser-known functions, the pharmacotherapies inhibiting its function in DNA repair, and its potential as a therapeutic target in a broader context.

## Introduction

The DNA-dependent protein kinase (DNA-PK) is a serine/threonine protein kinase consisting of a catalytic subunit (DNA-PKcs) and a Ku heterodimer that is made up of the Ku70 and Ku80 subunits. DNA-PK was accidentally discovered after researchers studying translation found that double-stranded DNA (dsDNA) contaminated their preparations, leading to the phosphorylation of specific proteins (1). Early work showed that DNA-PK phosphorylates Sp1 in the formation of Sp1 transcription complexes (2, 3). It was soon established that DNA-PK was involved in repairing double-strand breaks (DSBs) through non-homologous end-joining (NHEJ). Since then, DNA-PK's role in the DNA damage response (DDR) pathways has been expanded to include pathway choice between NHEJ and homologous recombination (HR) (4) and in the immune system through V(D)J and class-switch recombination (5). Given its critical function in DDR pathways, DNA-PK has been targeted in cancer therapy in concert with DNA-damaging agents (6). More recently, DNA-PK has been implicated in other cellular processes, including cell cycle progression (7) and telomere maintenance (Table 1) (33). These findings, combined with the transcriptional targets that associate with DNA-PK, suggest that DNA-PK is pivotal in pathways outside of the DDR that are critical to cellular survival and proliferation.

**Table 1:** Protein targets of DNA-PK and their associated cellular functions.

**Protein Target**	**Function**	**References**
**DNA DAMAGE RESPONSE**		
**Non-homologous end joining**		
Artemis	Contributes to end-processing of DSB	(8)
DNA-PKcs	Phosphorylates factors involved in NHEJ	(8–11)
H2AX	Retains factors involved in DSB repair	(12)
Ku70/Ku80	Binds to DNA, recruits components of NHEJ machinery	(8, 9)
XLF	Stabilizes ends of DSBs	(8, 9)
XRCC4	Stabilizes ends of DSBs and ligates DSB with Ligase IV	(8, 9)
**Homologous recombination**		
BRCA1	Inhibits DNA-PKcs-mediated autophosphorylation	(13)
DNA-PKcs	Involved in DDR pathway choice via differential phosphorylation	(14, 15)
H2AX	Retains factors involved in DSB repair	(12)
RPA	Promotes HR after phosphorylation via recruitment of Rad51	(16, 17)
**NON-DNA DAMAGE RESPONSE**		
**Cell cycle progression**		
Chk2	Forms complex with BRCA1 to organize mitotic spindle	
DNA-PKcs	Localizes to centrosomes and kinetochores	(18–20)
MDM2/HDM2	Overcomes p53-mediated G1 phase cell cycle arrest	(21, 22)
p53	Causes cell cycle arrest in G1 phase	
PLK1	Regulates mitotic entry	(19, 23)
PP6	Regulates mitotic exit	(23)
**Transcriptional regulation**		
AR	Drives expression of prostate cancer-associated genes	(24)
NRE	Impairs glucocorticoid-induced MMTV transcription	(25)
OCT1 & OCT2	Drive expression of genes in multiple tissues	(26)
RNA Pol I	Involved in transcriptional elongation	(27)
Sp1	Functions as general transcription factor	(2)
TBP	Functions as general transcription factor	(28)
TFIIB	Functions as general transcription factor	(28)
TRIM28	Activates RNA Polymerase II to activate transcriptional elongation	(27)
**Telomere maintenance**		
DNA-PKcs	Facilitates telomere end-capping	(29–31)
hnRNP-A1	Maintains telomeric overhangs and activates telomerase	(29)
Ku70/Ku80	Maintains telomere length	(32)

**Figure 1:**
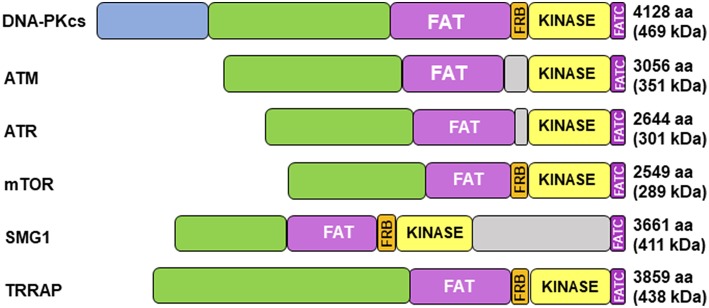
Structure of DNA-PKcs and related members of the phosphatidylinositol 3-kinase-related kinase (PIKK) family. DNA-PKcs can be subdivided into three large structural units: a large N-terminal helical domain, followed by the Circular Cradle, which contains multiple HEAT (Huntingtin, Elongation Factor 3, PP2A, and TOR1) repeats and a number of well-conserved phosphorylation clusters, and a C-terminal Head, which contains the highly conserved catalytic kinase domain. The kinase domain is flanked on either side by the well-conserved FAT (named for its homology in FRAP, ATM, and TRRAP) and FATC (FAT at the C-terminus) domains. The FKBP12-rapamycin-binding (FRB) domain, which sits between the FAT and kinase domain, is essential for mTOR kinase activity and subsequent G_1_ to S cell cycle progression, however, it may serve a different purpose in DNA-PK. The N-terminus contains HEAT repeats (blue) that make contact with other HEAT repeats (green). The FAT and FATC domains (purple) help stabilize the catalytic domain (yellow), which is adjacent to the FRB domain (orange). ATM, ataxia-telangiectasia mutated; ATR, ataxia telangiectasia and Rad3-related protein, mTOR, mammalian target of rapamycin; SMG1, one of the serine/threonine-protein kinases; TRRAP, transformation/transcription domain-associated protein.

Cloning of the cDNA of DNA-PKcs showed significant homology with the phosphatidylinositol 3-kinase (PI3K) family, however it did not have any activity toward lipids (34). At 460 kDa, DNA-PKcs is the largest of six serine/threonine kinases in the phosphatidylinositol 3-kinase-related kinase (PIKK) family, consisting of 4,128 amino acids (35). The PIKK family share significant homology (Figure 1) (36, 37). Ku heterodimerization is essential to maintain the stability of both subunits, loss of one subunit leads to decreased levels of the other (38). Although there is significant sequence divergence in the subunits in higher eukaryotes, especially compared to lower organisms, there is structural homology in both subunits (39). Ku70 and Ku80 contain three domains: an alpha helix/beta barrel von Willebrand A (vWA) domain on the N-terminus, a DNA-binding/dimerization core, and a helical domain at the C-terminus. The vWA domain functions as a surface for protein interactions, mediating binding between DNA-PK and factors involved in DNA repair, telomere regulation, and other functions (40). The C-terminal domain (CTD), where the majority of sequence divergence exists, contains the nuclear localization signal (NLS) on both subunits. Although Ku functions as a heterodimer, each subunit can independently import into the nucleus (38). The Ku70 CTD contains the SAP domain that increases the affinity of DNA-binding, whereas the Ku80 CTD houses the critical DNA-PKcs binding region (40).

## DNA-PK in DNA Repair

The role of DNA-PK in DNA repair has been extensively reviewed (41), and thus is briefly summarized here. Three main pathways exist to repair damaged DNA: classical NHEJ (C-NHEJ), alternative NHEJ (A-NHEJ), and HR. HR repairs DNA with the greatest fidelity because it uses sister chromatids to repair DSBs, but can only occur in the S and G2 phases of the cell cycle. Both NHEJ pathways can occur throughout the cell cycle, though A-NHEJ is more active during S phase (42). As opposed to the use of template strands in HR, NHEJ ligates two strands of DNA across a break. C-NHEJ is the primary form of DNA repair in higher eukaryotes, due to its simplicity and presence throughout the cell cycle. If C-NHEJ is unable to repair a DSB, the error-prone A-NHEJ becomes the dominant pathway (40, 43). But before a damage pathway is pursued, a cell must detect the presence of DSBs. H2A histone family member X (H2AX), is phosphorylated both by DNA-PKcs and ATM at its Ser139 residue to form γ-H2AX, a marker of DNA damage, that functions to retain factors involved in DSB repair (12).

### DNA-PK in Non-homologous End-Joining

By recruiting specific enzymes, NHEJ can repair DSBs of varying complexity, like those with incompatible ends or damaged bases (44). The sequence of NHEJ can be described as: (a) DSB end-recognition and binding by Ku; (b) assembly of the components of the NHEJ machinery, such as DNA-PKcs, the XRCC4-DNA ligase IV complex, and XRCC4-like factor (XLF); (c) activation of DNA-PKcs kinase activity; (d) bridging and, if necessary, end-processing of the broken DNA strands; (e) end-ligation by the XRCC4-DNA ligase IV complex; and (f) dissociation of the NHEJ machinery (36, 44, 45). The order of events following Ku binding to DNA is unknown; NHEJ is a dynamic process involving multiple factors interacting simultaneously.

In the first step of NHEJ, the Ku heterodimer recognizes and binds to the free ends of the DSB and recruits the canonical factors involved in NHEJ, including XRCC4-DNA ligase IV (9), XLF (46), and DNA-PKcs. Caspase-2-mediated cleavage of Ku80 at Asp726 may allow for DNA-PKcs binding and formation of the DNA-PK complex (47), causing an inward translocation of the Ku heterodimer and DNA-PKcs activation through conformational changes in the FAT and FATC domains (45). The DNA-PK complex likely tethers broken DNA strands, thereby preventing their nucleolytic degradation (48). DNA-PKcs phosphorylates members of the NHEJ machinery, including Ku, XRCC4, XLF, DNA-PKcs itself, and Artemis, which is involved in DNA end-processing (8). DNA-PKcs autophosphorylation at Thr2609 and Thr2647 in the ABCDE cluster mediates a conformational change in DNA-PKcs allowing for DNA end-processing (10). Conversely, mutagenesis of Ser2056, another known autophosphorylation site in the PQR cluster, showed that it likely limits end-processing (11).

While autophosphorylation appears to be crucial in NHEJ, the importance of binding interactions and DNA-PKcs-mediated phosphorylation of components of the NHEJ machinery remains unclear (49). Ku80 is crucial in immobilizing the broken ends of chromosomes within the nucleus, allowing for proper alignment at the site of repair (50). Cells harboring a deletion of the Ku80 carboxyl terminus showed increased sensitivity to IR and decreased levels DNA-PKcs autophosphorylation at Thr2647 when compared with controls, but levels of the autophosphorylated Ser2056 residue were unchanged (51). Mutant Ku heterodimers containing alanine instead of serine or threonine at residues 6, 577, and 580 of Ku70 and 715 of Ku80 were still able to function in DNA-damage repair (52).

### DNA-PK in Homologous Recombination

When faced with DNA damage-inducing stress, a cell has a choice between NHEJ and HR, but the competition between the two pathways is not fully understood. The availability of sister chromatids in the S and G2 phases of the cell cycle make HR a more favorable outcome, but some mechanism must exist to inhibit NHEJ, which can be activated at any point in the cell cycle. Breast Cancer 1, early onset (BRCA1), a canonical HR factor, functions in various capacities during DNA repair. In the context of pathway choice, BRCA1 prevents NHEJ in the S and G2 phases by inhibiting DNA-PKcs autophosphorylation at Ser2056. This interaction, mediated BRCA1's BRCT domain binding to DNA-PKcs, occurs in a phosphorylation-independent manner (13).

Beyond cell cycle considerations, other factors influencing the decision to pursue one DDR pathway over another remain unclear. Cells with inactivating mutations in Ku and DNA-PKcs will preferentially use HR as the primary DDR mechanism (14). Perhaps DNA repair pathway choice centers on whether DNA-PKcs is activated via phosphorylation: a phosphorylated/active form of DNA-PKcs favors NHEJ, while an unphosphorylated/inactive form favors HR. However, seemingly contradictory findings indicate a more nuanced mechanism. While mutagenesis and inactivation of DNA-PKcs that impaired NHEJ favored HR, pharmacological inhibition of DNA-PKcs impaired HR (15).

Replication protein A (RPA), a heterodimer that binds to single-stranded DNA (ssDNA), is an important modulator of HR. RPA complexes with tumor suppressor protein p53 and is hyperphosphorylated after DNA damage via DNA-PKcs (16). Coupled with the phosphorylation of p53, this hyperphosphorylation causes dissociation of the RPA-p53 complex and allows RPA to bind to ssDNA and promote HR via Rad51. Cells treated with camptothecin, followed by siRNA knockdown of DNA-PKcs-mediated phosphorylation of residues of RPA32, showed impaired HR (17).

## Functions of DNA-PK Outside of DNA Repair

Aside from its well-known role in two of the DDR pathways, DNA-PK functions in other cellular processes, such as cell cycle progression, transcription, and telomere maintenance. These functions may be involved in tumor progression, highlighting DNA-PK's potential as a therapeutic target.

### DNA-PK's Role in Cell Cycle Progression

Upon genotoxic stress, p53 causes cell cycle arrest in G1. Human/murine double minute 2 (MDM2; HDM2 in humans) overcome this blockade by complexing with p53. Since its discovery, MDM2—and its interplay with p53—has been targeted in cancer therapy (53). DNA-PKcs regulates this interaction by phosphorylating HDM2 at Ser17 to prevent binding with p53 (21). DNA-PK also acts on p53 by phosphorylating its Ser15 and Ser37 residues, inducing a conformational change that prevents HDM2 binding (22).

Cells are susceptible to DNA damage during S phase, which results in stalling or collapse of the replication fork. Replication stress leads to the formation of one-ended DSBs that are bound by RPA. Linked to its role in HR, DNA-PKcs-mediated phosphorylation of Ser4 and Ser8 of RPA32 causes growth arrest and delays mitotic entry (54).

DNA-PKcs has been implicated in the regulation of mitosis. Numerous studies have shown that reduction of DNA-PKcs enzymatic activity, either by pharmacological inhibition or by siRNA-mediated knockdown, leads to defects in chromosomal alignment and in nuclear morphology (7). Phosphorylation of DNA-PKcs at Ser2056, Thr2609, Thr2647, and Thr3950 causes DNA-PKcs localization to centrosomes. Phosphorylated Thr2609 is also seen at kinetochores during metaphase and cytokinesis (18, 19, 23). Phosphorylation at Thr2609 causes an association with polo-like kinase 1 (PLK1) in the mitotic phase, which regulates mitotic entry and exit, throughout mitosis at multiple subcellular structures. This interaction is essential for chromosomal segregation (19). Ser3205, another residue on DNA-PKcs that is likely essential for the overall success of mitosis, is phosphorylated by PLK1, allowing for the localization of DNA-PKcs to the midbody during cytokinesis. Dephosphorylation of Ser3205, via protein phosphatase 6 (PP6), occurs when cells exit mitosis (23), indicating that phosphorylation of this specific residue mediates mitotic entry and exit. DNA-PKcs also phosphorylates downstream targets involved in mitotic regulation. The Chk2-BRCA1 signaling pathway, which organizes the mitotic spindle, depends on DNA-PKcs activity. Chk2-mediated phosphorylation at Ser988 of BRCA1 ensures proper kinetochore-microtubule attachment. DNA-PKcs regulates Chk2 activity through the phosphorylation of its Thr68 residue. Knockdown of DNA-PKcs by siRNA inhibited phosphorylation of Thr68 on Chk2 and impaired microtubule growth during mitosis (55).

### DNA-PK as a Regulator of Transcription

Once established, the critical role DNA-PK plays in the DDR pathways became the dominant focus of its study. However, DNA-PK is critical for efficient gene expression, both in mediating transcriptional machinery and in modulating transcription factors. *In vitro*, Chinese hamster ovarian cells with a Ku70/Ku80 or DNA-PKcs deficiency showed a 2–7-fold decrease in transcription with multiple promoters compared to controls (56). RNA polymerase II activity requires functional activity of the TRIM28 factor, which is phosphorylated by DNA-PKcs at Ser824 (27). DNA-PK is involved in the phosphorylation of the general transcription factors TATA-binding protein (TBP) and transcription factor IIB (TFIIB), allowing them to synergistically form complexes with RNA polymerase and transcription factor IIF to stimulate basal transcription (28). The earliest defined role of DNA-PKcs in transcription was its activity on the transcription factor Sp1, which activates cellular promoters by binding to GC-rich regions. Upon binding to promoters, multiple residues of Sp1 are phosphorylated by DNA-PKcs (2). DNA-PKcs is also involved in the phosphorylation and activation of the POU domains of octamer-binding transcription factors 1 and 2 (OCT1 and OCT2) (26). Serine residues of c-MYC, the oncoprotein responsible for transcription of ~15% of the human genome (57), are phosphorylated by DNA-PKcs (58). DNA-PKcs also mediates the transcriptional activation of factors involved in metabolism. After feeding or in response to insulin, DNA-PK phosphorylates the upstream stimulatory factor-1 (USF-1) transcription factor at its Ser262 residue. The DNA-PK-USF complex induce transient breaks in the fatty acid synthase (FAS) promoter region immediately preceding transcriptional activation. Once transcribed, FAS can induce lipogenesis. DNA-PKcs-deficient mice fail to induce lipogenesis and are deficient in triglyceride levels (59). In 17β-estradiol (E_2_)-treated Michigan Cancer Foundation (MCF)-7 cells, topoisomerase IIβ-induced DSBs of the *pS2* promoter appear to be critical component of signal-dependent activation of gene transcription. These transient DSBs activate the enzymatic activity of poly(adenosine diphosphate-ribose) polymerase-1 (PARP-1). DNA-PKcs and the Ku heterodimer were copurified with PARP-1, suggesting that DNA-PK may be involved in transcriptional activation at these transient breaks (60). Recent studies demonstrated that DNA-PKcs functions in the progression of hormone-driven cancers. In advanced prostate cancer, DNA-PKcs coactivates the androgen receptor (AR), promoting metastatic phenotypes. Depletion of DNA-PKcs reduced expression of AR-regulated genes, delaying the formation of metastases (24). In breast cancer, DNA-PKcs-mediated phosphorylation of the estrogen receptor-α (ERα) at Ser118, leads to its stabilization and transcriptional activation. Inhibition of DNA-PK, either pharmacologically or via siRNA, reduced activation of ERα as well as increased its ubiquitination and subsequent degradation (61).

### DNA-PK and Telomere Maintenance

Given that telomeres are essentially endogenously occurring DSBs, it seems likely that DNA-PK would be intricately involved in their regulation. Paradoxically, DNA-PK's role in telomere maintenance is to protect against the processing and fusion associated with DSBs. The Ku70/Ku80 heterodimer has been implicated in several processes involving telomeres, including the silencing of telomere-proximal genes, tethering of telomeres to the nuclear periphery, and protecting telomeres from nucleolytic degradation (32, 62). Ku80 is critical for telomere length; siRNA-mediated knockdown of Ku80 led to significant telomere shortening (63). Similar results were produced in mice and human cells when DNA-PKcs activity was impaired (29, 33). Telomeric repeat-containing RNA (TERRA), a long non-coding RNA transcribed from telomeric DNA, has been implicated in processes related to telomere maintenance, such as the formation of heterochromatin (64, 65), replication (65), and end-capping (66). TERRA activity is thought to be mediated by the heterogenous nuclear ribonucleoprotein A1 (hnRNP A1). DNA-PKcs-mediated phosphorylation of hnRNP A1 removes TERRA from chromatin, allowing for telomere replication. Inhibition of DNA-PKcs/hnRNP A1 activity resulted in TERRA accumulation at telomeres, impairing efficient replication (66). DNA-PKcs is instrumental in facilitating telomere end-capping, likely through an interaction with the kinase interacting protein (KIP) and the telomeric repeat-binding factor 2 (TRF2), a subunit of the shelterin complex (67). Phosphorylation of Ser2056 of DNA-PKcs mediates end-capping. In its absence, uncapped telomeres are seen as DSBs and are processed, leading to inappropriate fusion events (30). Pharmacological inhibition of DNA-PKcs showed similar results in a concentration-dependent manner (31).

## DNA-PK and Cancer

Deregulated DNA-PK activity is associated with a number of cancers. In melanoma, DNA-PKcs acts as a metastatic driver by stimulating angiogenesis and tumor migration. DNA-PKcs activity was associated with the secretion of pro-metastatic proteins through modification of the tumor microenvironment (68). Increased expression and deregulation of DNA-PKcs was demonstrated to drive the development of hepatocellular carcinoma (69, 70). Upregulation of DNA-PKcs has also been observed in multiple myeloma (71), and, along with increased expression of the Ku subunits, is associated with radioresistance in cancers of the thyroid (72), nasopharynx (73), oral cavity (74), and cervix (75). Coupled with its critical cellular functions, these findings have made DNA-PK a prime therapeutic candidate in the treatment of malignancy.

## Pharmacotherapies Targeting DNA-PK

The development of DNA-PK inhibitors relied on earlier studies that synthesized small molecules PI3K inhibitors. Quercetin, a naturally occurring bioflavonoid, acted as a competitive antagonist against the kinase domain of PI3K and other protein and lipid kinases. This non-selectivity proved to be useful, as quercetin was used as a model compound to develop targeted inhibitors. 2-(4-Morpholinyl)-8-phenyl-4H-1-benzopyran-4-one (LY294002), was developed as a selective and competitive inhibitor of PI3K activity. Unlike quercetin, LY294002 had zero activity against other kinases and had a 2.7-fold increase in potency (IC_50_ = 1.5–2.0 μM) (76).

The specificity and potency of LY294002 against PI3K activity made it an ideal structural lead compound to develop new inhibitors that specifically target DNA-PKcs. This next generation of inhibitors, the 2,6-disubstituted pyran-4-one and thiopyran-4-one inhibitors, were more potent (IC_50_ = 1.1 and 0.72 μM, respectively) and selective for DNA-PKcs when compared to LY294002 (77). This led to the development of chromen-4-one derivatives, 2-*N*-morpholino-8-dibenzofuranyl-chromen-4-one (NU7427) and 2-*N*-morpholino-8-dibenzothiophenyl-chromen-4-one (NU7441). Compared to previous DNA-PKcs inhibitors, NU7427, and NU7441 were significantly potent (IC_50_ = 40 and 13 nM, respectively) and specific. At concentrations of 100 μM, NU7441 did not have an effect on ATM or ATR activity and showed minimal activity against PI3K and mTOR (78). NU7441 potentiates the effects of DNA damage-inducing chemotherapy in B-cell chronic lymphocytic leukemia (CLL) (79), breast (80), non-small cell lung carcinoma (NSCLC) (81), and nasopharyngeal carcinoma (NPC) (82) cell lines, as well increasing sensitivity to IR and chemotherapy in colorectal carcinoma cell lines (83). In an attempt to optimize the pharmacologic profile of NU7441, focused libraries were used to identify the biological activity of substitutions at the dibenzothiophene-1 position. The addition of water-soluble groups at this position proved to be effective, leading to the development of a new chromen-four-one derivative that has an even greater potency (IC_50_ = 6 nM). Unfortunately, this novel inhibitor may have some undesirable off-target effects (84). However, these findings highlight the ability to further modify known DNA-PKcs inhibitors, specifically with water-soluble groups, to develop more potent therapies. Another strategy to develop novel inhibitors was to use a homology model of the ATP-binding site of DNA-PK, based on the crystal structure of PI3Kγ. KU-0060648, a dual DNA-PKcs and PI3K inhibitor, has better bioavailability and a more favorable pharmacokinetic profile compared to NU7441 and also has limited activity against other PIKK family members. KU-0060648 is also more potent, with a 500-fold increase in solubility compared to NU7441 (85). DNA-PK inhibitors have also been demonstrated as effective single agents, taking advantage of synthetic lethality in ATM-deficient lymphoma models (86).

Another strategy taken to target DNA-PKcs activity in cancer is through the use of non-coding microRNAs (miRNAs). One study identified miR-101 as targeting both DNA-PKcs and ATM. Upregulation of miR-101 sensitized glioblastoma and non-small cell lung cancer cell lines to IR (87). Another study demonstrated that transfection with has-miR-96-5p and has-miR-874-3p combined with IR decreased survival of non-small cell lung cancer cell lines when compared to IR alone, and had a similar effect when compared to a DNA-PK inhibitor (NU7026) plus IR (88).

DNA-PK has been targeted with antibodies and inhibitors specific to the Ku heterodimer. Though antibodies are generally ineffective against intracellular targets, there has been success with ScFv 18-2, which conjugates with folate via a scissile disulfide linker and enters cells through folate receptor-mediated endocytosis. Lung cancer cell lines treated with ScFv 18-2 showed increased levels of γ-H2AX and decreased phosphorylation of Ser2056. Compared to controls, treated cell lines were more radiosensitive (89). Based on the crystal structure of the Ku70/Ku80 heterodimer (7-{[2-(3,4-dimethoxyphenyl)ethyl]amino}-3-(3-fluorophenyl)pyrimido[4,5-d]pyrimidine-2,4(1H,3H)-dione (Compound L), was developed as an inhibitor that disrupts the Ku heterodimer binding to DNA. Compound L decreased phosphorylation of Ser2056 and downstream DNA-PK targets in glioblastoma cell lines (90).

The promising effects of DNA-PK inhibitors to sensitize tumors to chemotherapy and radiation has led to their implementation in clinical trials. M3814 is being tested with radiotherapy in advanced solid tumors (NCT02516813). CC-122, a pleiotropic pathway modulator with activity against DNA-PK, is in Phase 1 trials studying its effects in multiple myeloma, advanced solid tumors, and non-Hodgkin's lymphoma (NCT01421524). CC-115, a dual DNA-PK and mTOR inhibitor, is in Phase 2 studies to determine its efficacy in glioblastoma (NCT02977780).

## Conclusions

Since its discovery, DNA-PK has proven to be an intriguing modulator of many cellular functions. Its instrumental role in regulating how cells respond to genotoxic insult has been the dominant focus of research. Though much has been discovered, key questions remain that will help elucidate DNA-PK's role in cancer. Are there other substrates of DNA-PK that are yet to be discovered? How does the activity of the Ku heterodimer and DNA-PKcs change in malignancy? Finally, can the specific interactions between DNA-PK and its many substrates be targeted? Thus, far, DNA-PK inhibitors have focused on potentiating DNA damage through inhibition of its kinase function, thereby blocking phosphorylation of key enzymes involved in DNA repair. But these therapies represent a small portion of the therapeutic strategies that may be implemented to target DNA-PK. Novel inhibitors that impair the protein-protein interactions between DNA-PK and its many substrates have the potential to be more targeted and potent. In order to develop this next generation of inhibitors, further study on the regions of DNA-PK that are crucial for substrate binding is warranted. Given the recent findings on its structural properties, the many functions and pathways it regulates, and its therapeutic potential, DNA-PK remains a subject of great importance that may contribute greatly to our overall understanding of cancer and to the discovery of novel therapeutics.

## Author Contributions

All authors listed have made a substantial, direct and intellectual contribution to the work, and approved it for publication.

### Conflict of Interest Statement

The authors declare that the research was conducted in the absence of any commercial or financial relationships that could be construed as a potential conflict of interest.
